# A Comparison of Muscle Sympathetic Nerve Activity to Non-contracting Muscle During Isometric Exercise in the Upper and Lower Limbs

**DOI:** 10.3389/fnins.2019.00341

**Published:** 2019-04-09

**Authors:** Daniel Boulton, Simon Green, Vaughan G. Macefield, Chloe E. Taylor

**Affiliations:** ^1^School of Medicine, Western Sydney University, Sydney, NSW, Australia; ^2^School of Science and Health, Western Sydney University, Sydney, NSW, Australia; ^3^Neuroscience Research Australia, Sydney, NSW, Australia; ^4^Human Autonomic Neurophysiology, Baker Heart and Diabetes Institute, Melbourne, VIC, Australia

**Keywords:** muscle contraction, metaboreflex, ischaemia, microneurography, muscle sympathetic nerve activity

## Abstract

Previous research indicates that greater sympathetic vasoconstrictor drive to skeletal muscle occurs during isometric upper limb exercise compared to lower limb exercise. However, potential disparity between blood flow and metaboreflex activation in contracting upper and lower limbs could contribute to the augmented sympathetic response during upper limb exercise. Therefore, the aim of this study was to examine MSNA responses during ankle dorsiflexion and handgrip exercise under ischaemic conditions, in order to standardize the conditions in terms of perfusion and metaboreflex activation. Eight healthy male subjects performed 4-min contractions of ischaemic isometric handgrip and ankle dorsiflexion at ∼10% maximal voluntary contraction, followed by 6 min of post-exercise ischaemia. MSNA was recorded continuously by microneurography of the common peroneal nerve of the non-contracting leg and quantified from negative-going sympathetic spikes in the neurogram, synchronized with the cardiac cycle. The time-course of MSNA exhibited parallel increases during exercise of the upper and lower limbs, rising throughout the contraction to peak at 4 min. This represented an increase of 100% relative to resting levels for handgrip exercise (66 ± 24 vs. 33 ± 7 spikes/min at rest) and 103% for dorsiflexion (63 ± 25 vs. 31 ± 8 spikes/min at rest; *P* < 0.01). In both conditions MSNA remained elevated during post-exercise ischaemia and returned to pre-contraction levels during recovery. These findings demonstrate that that the MSNA response to metaboreflex activation is similar for upper and lower limb exercise when perfusion is controlled for.

## Introduction

Muscle metaboreflex activation during sustained isometric exercise causes a progressive decrease in muscle vascular conductance (via sympathetically mediated vasoconstriction) and an increase in arterial pressure (Mitchell et al., 1983; Andersen and Saltin, 1985). Whilst there are reports of increases in muscle sympathetic nerve activity (MSNA) to inactive skeletal muscle in the arm (Saito et al., 1989; Seals and Enoka, 1989; Cui et al., 2001; Ichinose et al., 2004, 2006) and leg (Ray et al., 1992; Ray and Mark, 1993; Hansen et al., 1994) during exercise, the consistency of cardiovascular and sympathetic responses to exercise between different muscle groups is not clear. Ray et al. (1992) reported that MSNA is augmented during upper limb exercise compared to lower limb exercise at the same intensity. However, the isometric handgrip and knee extension exercise performed in this study involve vastly disparate muscle masses, which may account for the differential cardiovascular and sympathetic responses to exercise.

Saito (1995) compared MSNA responses to upper and lower limb exercise during isometric handgrip, dorsiflexion and plantarflexion, thus providing greater consistency with regards to muscle mass. They reported that the increases in MSNA were greatest during handgrip, followed by dorsiflexion and finally plantarflexion, which they attributed to differences in fiber type composition and the metabolic capacity associated with these fibers. The relative proportion of type II to type I fibers is greater in the forearm muscles compared with the tibialis anterior, and the proportion of type II is lower still in the soleus muscles (Johnson et al., 1973). Since type II fibers are highly glycolytic and fatiguable, muscles with greater proportions of these fibers may evoke a greater metaboreflex response and thus greater increases in MSNA. However, it should be noted that, when comparing responses between dorsiflexors and handgrip, there were no significant differences at the lowest (20% MVC) and highest (50% MVC) forces, but the MSNA response was significantly higher for handgrip at 33% MVC. These data suggest that the differences are not large and there is not a clear intensity-dependent effect. However, other influential factors, besides muscle mass and fiber type, may differ between upper and lower limbs during exercise. For instance, there is evidence to suggest that muscle blood flow during sustained contractions may be limited at different exercise intensities in the upper vs. lower limbs. During sustained contractions in the upper limb there is an increase in blood flow at intensities up to 70% MVC before blood flow is restricted (Kagaya and Homma, 1997). During sustained lower limb contractions, the increase in blood flow is limited to much lower intensities (Barcroft and Millen, 1939); Green et al. (2011) reported that hyperaemia was not present during sustained calf contractions exceeding 20% MVC. Given the lack of direct comparisons between upper and lower limb blood flow during sustained contractions, we cannot assume the blood flow is comparable even at lower exercise intensities. We therefore propose that contractions are performed whilst occluding blood flow to, firstly, standardize the conditions for the two limbs as much as possible (i.e., in terms of perfusion) and, secondly, to maximize metaboreflex activation. These conditions also provide the opportunity to use lower contraction intensities, during which a more stable MSNA recording can be maintained and contractions can be sustained for longer (4 min).

Both central command and the muscle metaboreflex have been implicated with the sympathetic and cardiovascular responses to sustained isometric exercise (Goodwin et al., 1972; Saito et al., 1989; Ray and Mark, 1993; Victor et al., 1995; Ichinose et al., 2006; Matsukawa, 2012). We have previously shown that central command is an important mechanism for mediating the sympathetic response to contracting muscle but has a negligible influence on MSNA to a non-contracting leg during exercise of the contralateral leg (Boulton et al., 2014, 2018). It is important to point out that these experiments consisted of isometric exercise; recent evidence suggests that central command may influence MSNA to non-contracting muscles during *isotonic* exercise, albeit attenuating a fall in total MSNA to non-contracting muscles through an increase in MSNA burst strength (Doherty et al., 2018). It is unclear whether metaboreflex-driven increases in MSNA to non-contracting muscles could respond differently to isometric contractions of the upper limb compared to isometric contractions of the lower limb. It is hypothesized that the MSNA response to metaboreflex activation does not differ between the upper and lower limb when contracting without perfusion. Therefore, the aim of the present study was to investigate sympathetic outflow to inactive leg muscles during ischaemic handgrip and dorsiflexion. A cuff was inflated to supra-systolic pressures around the upper arm (during handgrip) and thigh (during dorsiflexion). This expedites the build-up and turnover of metabolites stimulating group III/IV afferents, and standardizes the conditions in terms of perfusion. Sympathetic activity was measured from the negative-going sympathetic spikes in the raw neurogram (Bent et al., 2006; Fatouleh and Macefield, 2011, 2013; Hammam et al., 2011).

## Materials and Methods

### Participants

Experiments were performed on 11 healthy male subjects, aged 18 to 33 years, with no cardiorespiratory, metabolic or neuromuscular disease. Participants were instructed to abstain from alcohol consumption and vigorous exercise for 24 h prior to the study, and from caffeine on the day of the study. Individuals who smoked or took regular medication were excluded from participation in the study. The study was conducted with the approval of the Human Research Ethics committee, Western Sydney University, and in accordance with the Declaration of Helsinki. Participants provided written informed consent before taking part in the study. The data for the dorsiflexion condition have previously been presented in our study comparing MSNA responses to contracting vs. non-contracting muscles (Boulton et al., 2018).

### Measurements

Subjects were positioned semi-recumbent in a chair with their backs at 45°, legs supported horizontally and feet strapped in a plantarflexed position (95°) to independent footplates connected to a force transducer. Tungsten microelectrodes (Frederick Haer and Co., Bowdoinham, ME, United States) were used for microneurography to measure spontaneous MSNA from muscle fascicles of the left common peroneal nerve, located near the level of the fibular head, innervating the ankle dorsiflexor, ankle everter and toe extensor muscles; a reference microelectrode with an uninsulated tip was inserted approximately 1 cm from the active microelectrode. The common peroneal nerve was identified by electrical stimulation through a 2 mm diameter probe delivering a 0.2 ms pulse at 1 Hz with a 2–10 mA current (Stimulus Isolator, ADInstruments, Sydney, NSW, Australia). Further stimulation was used at a much lower current (1 mA) until twitches of the innervated muscle could be detected at 20 μA. Additional manipulation of the microelectrode was performed until specific observations were made: (i) electrical stimulation produced small contractions of the innervated muscle; (ii) increases of afferent discharges occurred upon passive stretching or tapping of the innervated muscle but not light stroking of the skin; (iii) clear, spontaneous bursts of MSNA were synchronized to the cardiac cycle; and (iv) a maximal inspiratory apnoea produced a sustained increase of spontaneous cardiac-locked bursts. Neural activity was amplified (gain 2 × 10^4^) and filtered (bandpass 0.3–5.0 kHz sampling) using an isolated amplifier and headstage (NeuroAmpEX, ADInstruments) and stored on computer (10 kHz sampling) using a computer-based data acquisition and analysis system (PowerLab 16SP hardware and LabChart, version 8; ADInstruments).

A single lead (II) electrocardiogram (0.3–1 kHz) was recorded with Ag-AgCl surface electrodes (BioAmp, PowerLab, ADInstruments) on the chest and sampled at 2 kHz. Respiration (DC – 100 Hz) was recorded using a strain gauge transducer (Pneumotrace II; UFI, Morro Bay, CA, United States) around the chest and sampled at 100 Hz. Continuous, non-invasive beat-to-beat blood pressure was measured at 400 Hz from the middle finger of the right hand using digital arterial plethysmography (Finometer Pro, Finapres Medical Systems, Enschede, Netherlands). An electromyogram (EMG) (10 Hz to 1 kHz) was recorded with Ag-AgCl surface electrodes over the tibialis anterior muscle and sampled at 2 kHz, which was normalized to the EMG during a maximal voluntary contraction (MVC). Force was measured using two load cells (Aluminum S Type EG PT) connected to two independent footplates, amplified (gain × 200, bandpass DC–10 Hz; Quad Bridge Amplifier; ADInstruments), sampled at 100 Hz and normalized to the MVC of the subject. To control blood flow to the right leg, a large (22 cm) sphygmomanometer cuff was wrapped around the right upper thigh and attached to a rapid cuff inflation system (AG101 and E20; Hokanson, Bellevue, WA, United States), which was set to a suprasystolic pressure (220 mmHg). Blood flow occlusion was verified by the absence of a pulse in the second toe, which was assessed using a piezoelectric pulse transducer (UFI, Morro Bay, CA, United States). A smaller cuff (13 cm) was wrapped around the right upper arm and attached to the rapid inflation system, with a switch to enable occlusion of blood flow to either the right arm or right leg. Again, absence of blood flow was confirmed by monitoring the pulse in a finger using the pulse transducer referred to above.

### Experimental Procedures

**FIGURE 1:**
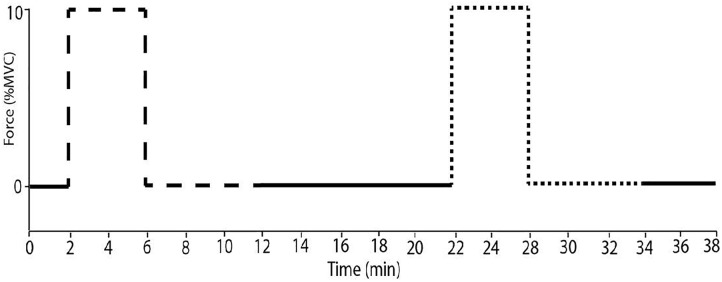
The experimental protocol. Subjects performed sustained ischaemic handgrip (dashed line) for 4 min at 10% MVC with ischaemia imposed for 6 min after contraction. Sustained dorsiflexion (dotted line) was performed under the same conditions with a minimum of 10 min of rest.

Maximal voluntary contraction of the right ankle (dorsiflexion) and right forearm (handgrip) was determined from two three-second attempts under freely perfused conditions. The experimental protocol began once a continuous MSNA recording was achieved. An initial 5-min baseline period was recorded to obtain resting measures of cardiovascular and sympathetic variables. In a random order, subjects performed one sustained isometric ankle dorsiflexion and one period of handgrip exercise, both at 10% MVC for 4 min. Ischaemia was imposed approximately 5 s before the onset of contraction and continued for 6 min post-contraction (Figure 1). Ten minutes of rest separated each contraction and the protocol was concluded with a 5-min rest period. To test the effect of ischaemia alone, an additional 5-min period of ischaemic rest was recorded at the end.

### MSNA Analysis

**FIGURE 2:**
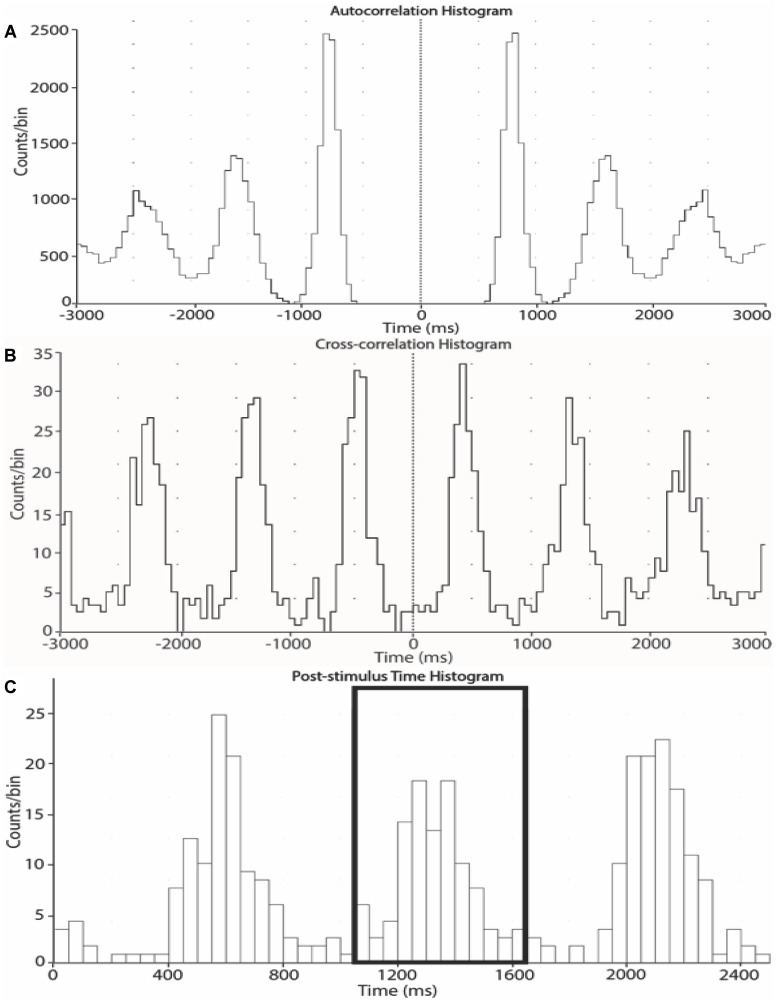
Autocorrelation **(A)**, cross-correlation **(B)** and post-stimulus time **(C)** histograms from one subject. The histograms were generated from 1-min epochs to measure the timing of neural spikes relative to cardiac beats and count the number of sympathetic spikes during each epoch.

Negative spikes (half width 0.2–0.6 ms) were clearly observed in the neurogram during contractions and detected using window discriminator software (Spike Histogram, LabChart 2.5, ADInstruments). To account for the delay between the R-wave (ECG) and MSNA in the peroneal nerve (Fagius and Wallin, 1980), the neurogram was shifted back in time (∼1.15–1.30 s) relative to the R wave. As previously described (Boulton et al., 2016), autocorrelation histograms for the cardiac signal were generated in 50 ms bins. Cross-correlation and post-stimulus time histograms were generated between negative spikes and R-R intervals, ensuring that robust cardiac modulation of spike counts was apparent. Minimal changes in heart rate occurred during contraction (see section “Results”). However, to ensure that any differences in spike counts between contraction and rest could be attributed to changes in MSNA burst intensity or incidence as opposed to heart rate, an equal number of cardiac cycles were used for the analysis of contraction and rest periods for each subject (mean number of cardiac cycles used = 68 ± 12). MSNA spike counts were measured in 1-min epochs, meaning that the number of cardiac cycles for the first minute of rest were determined, and the same number of cardiac cycles were used from each subsequent 1-min epoch. MSNA spike counts included the number of spikes during 600 ms periods after each R-wave (i.e., diastole) and which centered about a peak spike count. Autocorrelation, cross-correlation and post-stimulus time histograms between the MSNA and R-R intervals of the electrocardiogram were sampled in 50 ms bins to determine the cardiac modulation and verify the sympathetic origin of the selected spikes (Figure 2).

### Statistical Analysis

A two-way repeated measures ANOVA was performed to test for main effects and interactions between “time” and “exercising limb” (upper vs. lower limb) (Prism 5.0, GraphPad Software, United States). When a significant main effect was found by two-way ANOVA, the Sidak *post hoc* test was performed to make pairwise multiple comparisons between the last minute of rest prior to contraction and each of the subsequent minutes in the protocol. Significance was set at *P* < 0.05 and results are expressed as mean ± SD.

## Results

### Resting MSNA

**FIGURE 3:**
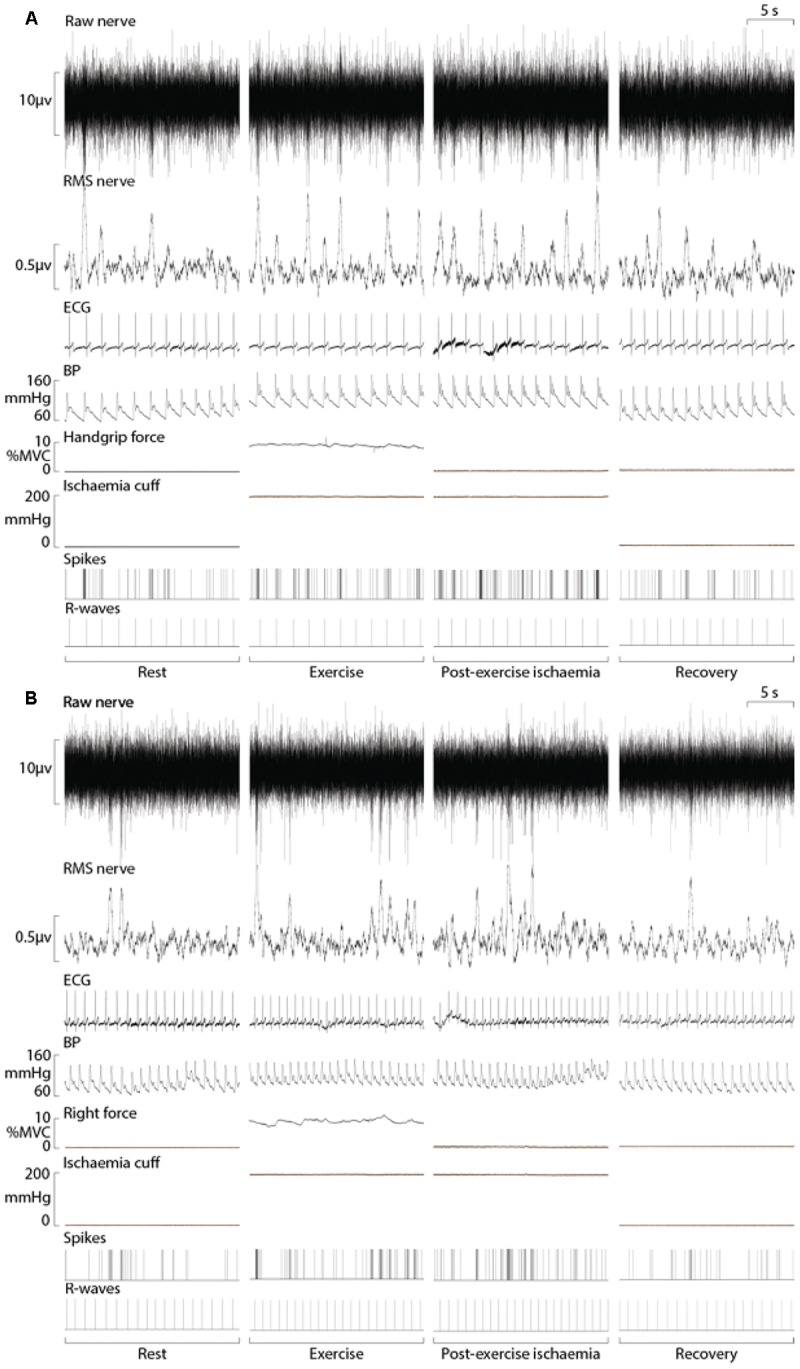
Experimental records from one subject during ischaemic handgrip **(A)** and ankle dorsiflexion **(B)** with a period of post-exercise ischaemia. Analysis of MSNA involved measuring the negative-going sympathetic spikes of the raw nerve signal.

Sympathetic recordings of negative-going spikes from one subject during an ischaemic isometric dorsiflexion of the right ankle (A) and an ischaemic isometric handgrip performed on the right side (B) are shown in Figure 3. The total number of sympathetic spikes for each 1-min epoch was measured from the post-stimulus time histogram of successful recordings from eight subjects. A rest period of 5 min before and after the protocol demonstrated a stable sympathetic signal, with MSNA being similar in each period (31 ± 9 vs. 29 ± 10 spikes/min; *P* = 0.50). At the end of the protocol a period of ischaemia without contraction showed no effect on MSNA (29 ± 9 spikes/min; *P* = 0.56) when compared with the initial 5-min rest period. MSNA in the minute of rest immediately prior to contraction were not significantly different for upper vs. lower limb exercise, with 33 ± 7 spikes/min prior to handgrip and 31 ± 8 spikes/min prior to dorsiflexion (*P* = 0.40).

### Effect of Upper Versus Lower Limb Exercise on MSNA

Experimental results for MSNA during ankle dorsiflexion and handgrip are shown in Figure 3. There was a significant main effect of time on MSNA (*P* < 0.01, *F* = 20.33), but no main effect of exercising limb (*P* = 0.42, *F* = 0.74) or significant interaction (*P* = 0.72, *F* = 0.76). For both handgrip and ankle dorsiflexion, MSNA increased incrementally for each minute of contraction, peaking at 63 ± 25 spikes/min for ankle dorsiflexion and 66 ± 24 spikes/min for handgrip, an increase of 103 and 100% of resting levels, respectively. In both conditions, MSNA remained elevated for the duration of post-exercise ischaemia and, upon cessation of circulatory arrest, returned toward pre-contraction levels.

### Cardiovascular Responses

**FIGURE 4:**
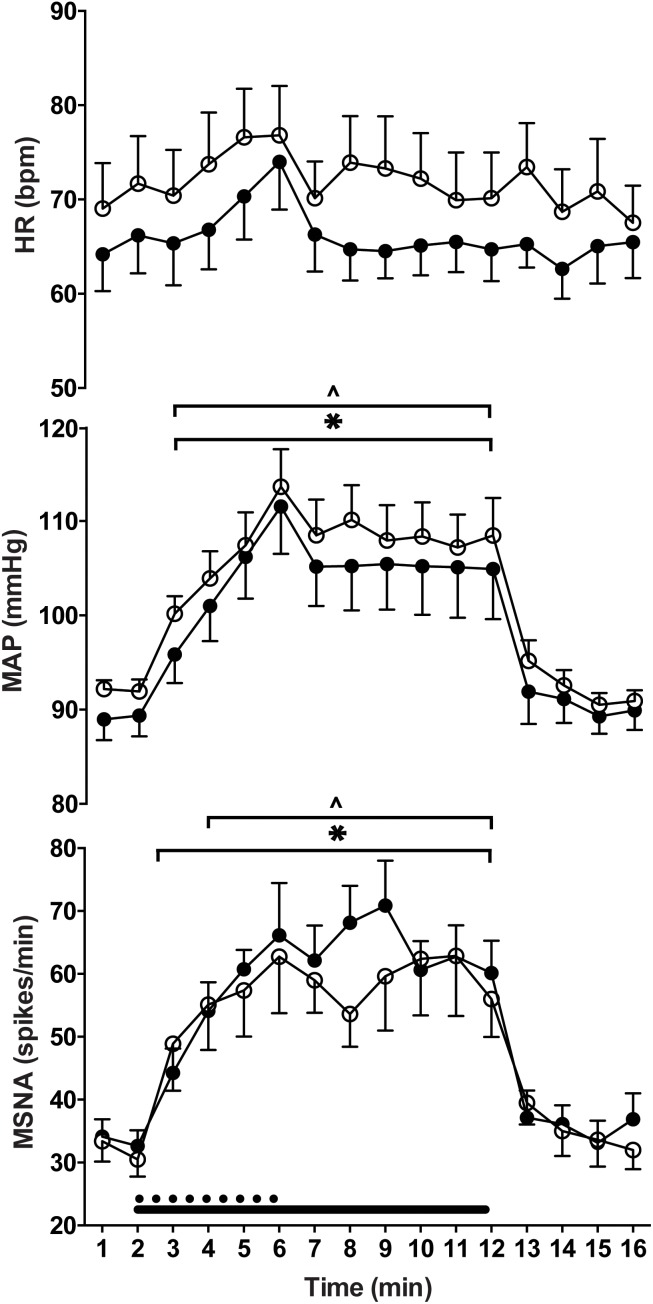
Heart rate (HR), mean arterial pressure (MAP) and muscle sympathetic nerve activity (MSNA) responses to contraction of the upper (filled circles) and lower (empty circles) limbs. There were significant increases from rest (last min of rest period preceding contraction) during contraction of the lower (^∗^) and upper limbs (ˆ) (*P* ≤ 0.05).

By design, there were no significant differences between the relative force outputs for handgrip and ankle dorsiflexion (*P* = 0.11) and no main effects of “time” or “limb” (upper vs. lower limb exercise) on respiratory rate (*P* > 0.05). Mean arterial blood pressure and heart rate responses were similar during ischaemic ankle dorsiflexion and handgrip (Figure 3). There was a significant effect of time (*P* < 0.01, *F* = 26.24), but not exercising limb (*P* = 0.23, *F* = 1.73) on mean arterial pressure. During both handgrip and dorsiflexion, blood pressure significantly increased during the first minute of contraction (*P* < 0.03) and continued to increase for each subsequent minute. In both conditions, the period of post-exercise ischaemia was associated with a small drop in blood pressure, but it remained elevated above pre-contraction levels (*P* < 0.01) until the cessation of ischaemia. Figure 4 indicates a gradual increase in heart rate during dorsiflexion and handgrip exercise, but this was not significant (*P* > 0.21). Heart rate remained at resting levels for the duration of post-exercise ischaemia and recovery after dorsiflexion and handgrip.

## Discussion

In this study MSNA was measured to non-contracting muscles during sustained dorsiflexion of the ankle and sustained handgrip exercise, both performed in the presence of occlusion of blood flow to the contracting muscle and followed by a period of post-exercise ischaemia. The ischaemic nature of the contractions enhanced the excitation of metaboreceptors during these low intensity contractions and controlled for the potentially confounding factor of perfusion. The results revealed a similar incremental increase in MSNA during contractions of the upper and lower limbs. MSNA was elevated for the duration of post-exercise ischaemia and returned to pre-contraction levels upon cessation of ischaemia. The incremental rise in MSNA throughout the contraction indicates that the muscle metaboreflex may be the dominant mechanism involved in driving sympathetic outflow to non-contracting muscles, a conclusion we had reached in earlier studies (Boulton et al., 2014, 2016, 2018). The present findings demonstrate that sympathetic responses during isometric contractions of the upper and lower limbs are similar when controlling for potential differences in blood flow and muscle metaboreflex activation.

### MSNA During Upper Versus Lower Limb Contraction

Prior to the current study the behavior of sympathetic outflow to sustained contractions of the upper and lower limbs was not clear. Ray et al. (1992) reported an increase in MSNA during moderate-intensity handgrip (2 min at 30% MVC) but a decrease in MSNA during the first minute of knee extension at the same intensity, followed by an increase during the second minute. With such disparity in muscle mass we might expect to observe these differences in cardiovascular and sympathetic responses to exercise. For instance, Seals (1989) reported augmented sympathetic and cardiovascular responses to a larger active muscle mass during two-arm handgrip compared to single-arm handgrip. However, the observations by Seals (1989) could also be a product of increasing the number of simultaneously active limbs, both with respect to central command to, and reflex inputs from, the contracting muscles. The size of the muscle groups used in upper and lower limb exercise in the current study are more comparable, and therefore discrepancies in muscle mass are less likely to influence the results. Certainly, the parallel cardiovascular and sympathetic responses in the current study indicate that the forearm and pretibial flexor muscles produced the same level of work for each contraction.

Differential sympathetic responses to exercise of the upper and lower limbs observed in previous studies could be due to differences in intramuscular blood flow during exercise of the upper and lower limbs. Previous research suggests that blood flow through contracting muscle is restricted at significantly lower exercise intensities during lower limb exercise compared with exercise of the upper limb (Barcroft and Millen, 1939; Green et al., 2011). The metaboreflex represents a response to a mismatch between blood flow and metabolic demand, whereby inadequate blood flow during exercise leads to the build-up of metabolites produced during contraction and a reflex increase in arterial pressure (Alam and Smirk, 1937; Rowell et al., 1976; O’Leary and Joyner, 2006). Given that the blunting of hyperaemia may occur at lower exercise intensities in the leg vs. arm, it is conceivable that this mismatch occurs more readily in the lower limbs, thereby leading to an earlier activation of metaboreceptors. Although Green et al. (2011) demonstrated that hyperaemia may be blunted in the lower limbs at intensities of 20% MVC, we cannot rule out potential limb-dependent differences in blood flow and therefore possible disparities in metaboreflex activation between upper and lower limbs. Given that previous studies have indicated that MSNA responses to upper and lower limb exercise differ when blood flow is not experimentally occluded (Saito, 1995), our findings suggest that perfusion may have a role to play in the level of metaboreflex activation between limbs. However, if hyperaemia is blunted at lower intensities in the lower limb vs. upper limb, we might expect greater metaboreflex activation and thus greater MSNA responses to have been reported for lower limb exercise. Saito (1995), however, reported greater MSNA responses to handgrip exercise compared with dorsiflexion, albeit these differences were significant only for contractions at 33% MVC and not 20 or 50%. Our data therefore suggests that the effect observed by Saito (1995) may not be related to fiber type *per se* but is related to perfusion and its effect on susceptibility to fatigue. Saito et al. (1989) showed in a separate study that the sensation of fatigue in the working muscles during contraction is correlated with the increase in MSNA. Although type II fibers are highly fatigable, and thus muscles with greater proportions of these fibers may evoke a greater MSNA response, the fatigability of different fiber types are more similar when the muscles are deprived of blood and oxygen. This may explain the lack of differences in the MSNA responses in the current study. Specifically, we demonstrate that when the level of metaboreflex activation is controlled for, the MSNA responses to that activation are similar for muscles of the upper and lower limbs.

### Metaboreflex Control of MSNA

The failure of MSNA to increase during contraction in previous studies (Ray et al., 1992; Saito, 1995) could be due to an inadequate exercise stimulus (intensity and/or duration) (Ray and Mark, 1993) and metaboreflex activation. The contractions performed in the studies by Ray et al. (1992) and Saito (1995) were moderate to high-intensity (20–50% MVC) and of relatively short duration (2 min). The exercise parameters in the current study (ischaemic, low-intensity, sustained contractions) were implemented to reduce the influence of perfusion, while ensuring the metaboreflex was activated. Occlusion of blood flow to the active limb at the onset of contraction increases the concentration of metabolites, thus maximizing the stimulus for metaboreflex activation, as demonstrated by the sustained elevation in blood pressure during contraction and post-exercise ischaemia. In the present study, MSNA increased by 33% of resting levels during the first minute of ischaemic handgrip and by 58% of resting levels during the first minute of ankle dorsiflexion. These prompt increases in MSNA to contraction are quicker than observed in previous studies (Saito et al., 1986, 1989; Wallin et al., 1989; Ray et al., 1992; Ray and Mark, 1993; Saito, 1995; Cui et al., 2001; Ichinose et al., 2006) possibly because metabolic by-products were trapped from the onset of contraction, causing a more rapid increase in metabolite accumulation and a decrease in muscle acidity (pH) (Victor et al., 1988).

Numerous studies have indicated that central command does not directly contribute to the exercise-induced response of MSNA to non-active muscle (Victor et al., 1989; Wallin et al., 1992; Hansen et al., 1994), but rather exerts an influence on baroreflex resetting and vagal outflow to the heart during exercise (Victor et al., 1987; Cui et al., 2001; Querry et al., 2001; Matsukawa et al., 2005). However, the potential influence of central command on MSNA to an inactive limb cannot be dismissed because MSNA increased within 1 min of the start of exercise. Evidence suggests it has a role to play in isotonic exercise: Doherty et al. (2018) reported that the fall observed in total MSNA to inactive limbs during one-legged cycling is partially offset by an increase in MSNA burst strength driven by central command.

### Methodological Considerations

The rationale for occluding blood flow both during and after exercise was to eliminate the potentially confounding factor of limb blood flow and to control for metaboreflex activation by attempting to maximize activation in both limbs studied. Whilst this does allow us to examine the intrinsic muscle response to metaboreflex activation, it is acknowledged that the current study is limited by a lack of an exercise condition without occlusion, which would have allowed confirmation of such differences in MSNA responses between limbs. In addition, it is possible that exercise training status may influence metaboreflex activation (Sinoway et al., 1996) and, whilst we did not recruit participants based on their training history, we did collect self-reported activity levels. These reports indicate that the participants were typically involved in combinations of aerobic and resistance training of the upper and lower limbs (>2 times per week). Future research may be directed toward examining the effects of training history on metaboreflex activation through comparisons between sedentary, aerobic and resistance trained individuals.

We appreciate that presenting traditional MSNA burst analysis may have allowed for comparisons with other studies in the literature. However, we have used spike analysis for a number of reasons. Firstly, for consistency with our previous studies in which this approach was necessary due to the fact that EMG can infiltrate the MSNA signal, as can the activity of muscle spindle and Golgi tendon organ afferents and alpha motor axons when recording MSNA to the contracting limb. Secondly, the spike analysis is a more sensitive approach for capturing a greater proportion of the sympathetic nerve activity, because the negative-going spikes extracted from the neurogram are defined as sympathetic because of their strong cardiac rhythmicity. Conversely, the traditional burst analysis may suffer from a poor signal-to-noise ratio and baseline shifts due to the participant tensing leg muscles, leading to periods of EMG infiltration. This can significantly hinder the ability to reliably identify bursts and measure their amplitude.

## Conclusion

Metabolically mediated reflex control of sympathetic outflow to non-active skeletal muscle is essentially important for the regulation of blood pressure during movement and exercise. By controlling for differences in metaboreflex activation, our results support differences in metaboreflex activation as a mechanism for differences observed between upper and lower limb exercise in previous studies. Our findings suggest that the MSNA response is similar for upper and lower limb exercise, indicating that what actually differs is the level of metaboreflex activation when these muscles are “freely perfused.” Intrinsically, muscles do not differ in terms of the impact they have on MSNA; our data suggest that perfusion may be more important.

## Ethics Statement

The study was conducted with the approval of the Human Research Ethics committee, Western Sydney University, and in accordance with the Declaration of Helsinki. Participants provided written informed consent before taking part in the study.

## Author Contributions

Experiments were performed in the School of Medicine (Western Sydney University). All authors were involved in the design of the experiments and/or data acquisition and analysis of the data, as well as the writing or editing of this manuscript, and approved the final version of this manuscript and agreed to be accountable for all aspects of the work.

## Conflict of Interest Statement

The authors declare that the research was conducted in the absence of any commercial or financial relationships that could be construed as a potential conflict of interest.

## References

[B1] Alam M. Smirk F. H. (1937). Observations in man upon a blood pressure raising reflex arising from the voluntary muscles. *J. Physiol.* 89 372–383. 10.1113/jphysiol.1937.sp003485 16994867 PMC1395054

[B2] Andersen P. Saltin B. (1985). Maximal perfusion of skeletal muscle in man. *J. Physiol.* 366 233–249. 10.1113/jphysiol.1937.sp003485 4057091 PMC1193029

[B3] Barcroft H. Millen J. L. E. (1939). The blood flow through muscle during sustained contraction. *J. Physiol.* 97 17–31. 10.1113/jphysiol.1939.sp003789 16995147 PMC1393980

[B4] Bent L. R. Bolton P. S. Macefield V. G. (2006). Modulation of muscle sympathetic bursts by sinusoidal galvanic vestibular stimulation in human subjects. *Exp. Brain Res.* 174 701–711. 10.1007/s00221-006-0515-6 16721608

[B5] Boulton D. Taylor C. E. Green S. Macefield V. G. (2018). The metaboreflex does not contribute to the increase in muscle sympathetic nerve activity to contracting muscle during static exercise in humans. *J. Physiol.* 596 1091–1102. 10.1113/JP275526 29315576 PMC5851889

[B6] Boulton D. Taylor C. E. Macefield V. G. Green S. (2014). Effect of contraction intensity on sympathetic nerve activity to active human skeletal muscle. *Front. Physiol.* 5:9. 10.3389/fphys.2014.00194 24917823 PMC4042086

[B7] Boulton D. Taylor C. E. Macefield V. G. Green S. (2016). Contributions of central command and muscle feedback to sympathetic nerve activity in contracting human skeletal muscle. *Front. Physiol.* 7:163. 10.3389/fphys.2016.00163 27242537 PMC4865629

[B8] Cui J. Wilson T. E. Shibasaki M. Hodges N. A. Crandall C. G. (2001). Baroreflex modulation of muscle sympathetic nerve activity during posthandgrip muscle ischemia in humans. *J. Appl. Physiol.* 91 1679–1686. 10.1152/jappl.2001.91.4.1679 11568150

[B9] Doherty C. J. Incognito A. V. Notay K. Burns M. J. Slysz J. T. Seed J. D. (2018). Muscle sympathetic nerve responses to passive and active one-legged cycling: insight into the contributions of central command. *Am. J. Physiol. Heart Circul. Physiol.* 314 H3–H10. 10.1152/ajpheart.00494.2017 28939650 PMC5866391

[B10] Fagius J. Wallin B. G. (1980). Sympathetic reflex latencies and conduction velocities in normal man. *J. Neurol. Sci.* 47 433–448. 10.1016/0022-510X(80)90098-2 7420119

[B11] Fatouleh R. Macefield V. G. (2011). Respiratory modulation of muscle sympathetic nerve activity is not increased in essential hypertension or chronic obstructive pulmonary disease. *J. Physiol.* 589 4997–5006. 10.1113/jphysiol.2011.210534 21844003 PMC3224888

[B12] Fatouleh R. Macefield V. G. (2013). Cardiorespiratory coupling of sympathetic outflow inhumans: a comparison of respiratory and cardiac modulation of sympathetic nerve activity to skin and muscle. *Exp. Physiol.* 98 1327–1336. 10.1113/expphysiol.2013.072421 23625953

[B13] Goodwin G. M. McCloskey D. I. Mitchell J. H. (1972). Cardiovascular and respiratory responses to changes in central command during isometric exercise at constant muscle tension. *J. Physiol.* 226 173–190. 10.1113/jphysiol.1972.sp009979 4263680 PMC1331159

[B14] Green S. Thorp R. Reeder E. J. Donnelly J. Fordy G. (2011). Venous occlusion plethysmography versus Doppler ultrasound in the assessment of leg blood flow during calf exercise. *Eur. J. Appl. Physiol.* 111 1889–1900. 10.1007/s00421-010-1819-6 21234593

[B15] Hammam E. James C. Dawood T. Macefield V. G. (2011). Low-frequency sinusoidal galvanic stimulation of the left and right vestibular nerves reveals two peaks of modulation in muscle sympathetic nerve activity. *Exp. Brain Res.* 213 507–514. 10.1007/s00221-011-2800-2 21800255

[B16] Hansen J. Thomas G. D. Jacobsen T. N. Victor R. G. (1994). Muscle metaboreflex triggers parallel sympathetic activation in exercising and resting human skeletal muscle. *Am. J. Physiol. Heart Circ. Physiol.* 266 H2508–H2514. 10.1152/ajpheart.1994.266.6.H2508 8024012

[B17] Ichinose M. Saito M. Kondo N. Nishiyasu T. (2006). Time-dependent modulation of arterial baroreflex control of muscle sympathetic nerve activity during isometric exercise in humans. *Am. J. Physiol. Heart Circ. Physiol.* 290 H1419–H1426. 10.1152/ajpheart.00847.2005 16284234

[B18] Ichinose M. Saito M. Wada H. Kitano A. Kondo N. Nishiyasu T. (2004). Modulation of arterial baroreflex control of muscle sympathetic nerve activity by muscle metaboreflex in humans. *Am. J. Physiol. Heart Circ. Physiol.* 286 H701–H707. 10.1152/ajpheart.00618.2003 14715501

[B19] Johnson M. Polgar J. Weightman D. Appleton D. (1973). Data on the distribution of fibre types in thirty-six human muscles: an autopsy study. *J. Neurol. Sci.* 18 111–129. 10.1016/0022-510X(73)90023-3 4120482

[B20] Kagaya A. Homma S. (1997). Brachial arterial blood flow during static handgrip exercise of short duration at varying intensities studied by a doppler ultrasound method. *Acta Physiol. Scand.* 160 257–265. 10.1046/j.1365-201X.1997.00158.x 9246389

[B21] Matsukawa K. (2012). Central command: control of cardiac sympathetic and vagal efferent nerve activity and the arterial baroreflex during spontaneous motor behaviour in animals. *Exp. Physiol.* 97 20–28. 10.1113/expphysiol.2011.057661 21984731

[B22] Matsukawa K. Komine H. Nakamoto T. Murata J. (2005). Central command blunts sensitivity of arterial baroreceptor-heart rate reflex at onset of voluntary static exercise. *Am. J. Physiol. Heart Circ. Physiol.* 290 H200–H208. 10.1152/ajpheart.00013.2005 16113070

[B23] Mitchell J. H. Kaufman M. P. Iwamoto G. A. (1983). The exercise pressor reflex: its cardiovascular effects, afferent mechanisms, and central pathways. *Annu. Rev. Physiol.* 45 229–242. 10.1146/annurev.ph.45.030183.001305 6342515

[B24] O’Leary D. S. Joyner M. J. (2006). Counterpoint: the muscle metaboreflex does not restore blood flow to contracting muscles. *J. Appl. Physiol.* 100 357–361. 10.1152/japplphysiol.01222.2005 16402418

[B25] Querry R. G. Smith S. A. Stromstad M. Ide K. Raven P. B. Secher N. H. (2001). Neural blockade during exercise augments central command’s contribution to carotid baroreflex resetting. *Am. J. Physiol. Heart Circ. Physiol.* 280 H1635–H1644. 10.1152/ajpheart.2001.280.4.H1635 11247774

[B26] Ray C. A. Mark A. L. (1993). Augmentation of muscle sympathetic nerve activity during fatiguing isometric leg exercise. *J. Appl. Physiol.* 75 228–232. 10.1152/jappl.1993.75.1.228 8376268

[B27] Ray C. A. Rea R. F. Clary M. P. Mark A. L. (1992). Muscle sympathetic nerve responses to static leg exercise. *J. Appl. Physiol.* 73 1523–1529. 10.1152/jappl.1992.73.4.1523 1447100

[B28] Rowell L. B. Hermansen L. Blackmon J. R. (1976). Human cardiovascular and respiratory responses to graded muscle ischemia. *J. Appl. Physiol.* 41 693–701. 10.1152/jappl.1976.41.5.693 993157

[B29] Saito M. (1995). Differences in muscle sympathetic nerve response to isometric exercise in different muscle groups. *Eur. J. Appl. Physiol. Occup. Physiol.* 70 26–35. 10.1007/BF00601805 7729435

[B30] Saito M. Mano T. Abe H. Iwase S. (1986). Responses in muscle sympathetic nerve activity to sustained hand-grips of different tensions in humans. *Eur. J. Appl. Physiol. Occup. Physiol.* 55 493–498. 10.1007/BF00421643 3769906

[B31] Saito M. Mano T. Iwase S. (1989). Sympathetic nerve activity related to local fatigue sensation during static contraction. *J. Appl. Physiol.* 67 980–984. 10.1152/jappl.1989.67.3.980 2793727

[B32] Seals D. R. (1989). Influence of muscle mass on sympathetic neural activation during isometric exercise. *J. Appl. Physiol.* 67 1801–1806. 10.1152/jappl.1989.67.5.1801 2600014

[B33] Seals D. R. Enoka R. M. (1989). Sympathetic activation is associated with increases in EMG during fatiguing exercise. *J. Appl. Physiol.* 66 88–95. 10.1152/jappl.1989.66.1.88 2917961

[B34] Sinoway L. Shenberger J. Leaman G. Zelis R. Gray K. Baily R. (1996). Forearm training attenuates sympathetic responses to prolonged rhythmic forearm exercise. *J. Appl. Physiol.* 81 1778–1784. 10.1152/jappl.1996.81.4.1778 8904599

[B35] Victor R. G. Bertocci L. A. Pryor S. L. Nunnally R. L. (1988). Sympathetic nerve discharge is coupled to muscle pH during exercise in humans. *J. Clin. Invest.* 82 1301–1305. 10.1172/JCI113730 3170747 PMC442683

[B36] Victor R. G. Pryor S. L. Secher N. H. Mitchell J. H. (1989). Effects of partial neuromuscular blockade on sympathetic nerve responses to static exercise in humans. *J. Am. Heart Assoc.* 65 468–476. 10.1161/01.RES.65.2.468 2752552

[B37] Victor R. G. Seals D. R. Mark A. L. (1987). Differential control of heart rate and sympathetic nerve activity during dynamic exercise. Insight from intraneural recordings in humans. *J. Clin. Invest.* 79 508–516. 10.1172/JCI112841 3805279 PMC424115

[B38] Victor R. G. Secher N. H. Lyson T. Mitchell J. H. (1995). Central command increases muscle sympathetic nerve activity during intense intermittent isometric exercise in humans. *Circ. Res.* 76 127–131. 10.1161/01.RES.76.1.127 8001270

[B39] Wallin B. G. Burke D. Gandevia S. C. (1992). Coherence between the sympathetic drives to relaxed and contracting muscles of different limbs of human subjects. *J. Physiol.* 455 219–233. 10.1113/jphysiol.1992.sp019298 1484355 PMC1175641

[B40] Wallin B. G. Victor R. G. Mark A. L. (1989). Sympathetic outflow to resting muscles during static handgrip and postcontraction muscle ischemia. *Am. J. Physiol.* 256 H105–H110. 10.1152/ajpheart.1989.256.1.H105 2912172

